# The complete chloroplast genome sequence of *Tainia cordifolia* (Orchidaceae)

**DOI:** 10.1080/23802359.2019.1693304

**Published:** 2019-12-09

**Authors:** Qing-Dong Zheng, Jie Zhou, Shan-Hu Ma, Ming-Kun Chen, Tai-Xiang Xie, Juan Chen, Ye Ai

**Affiliations:** Key Laboratory of National Forestry and Grassland Administration for Orchid Conservation and Utilization, College of Landscape Architecture, Fujian Agriculture and Forestry University, Fuzhou, China

**Keywords:** *Tainia cordifolia*, chloroplast genome, Orchidaceae, phylogenetic analysis

## Abstract

*Tainia cordifolia* is a subtropical plant with significant ornamental value. Herein, we determined the complete chloroplast (cp) genome sequence of *T. cordifolia* using Illumina sequencing data. The whole cp genome is 158,089 bp in size, consisting of a pair of inverted repeats (IR 25,260 bp), a large single-copy region (LSC 86,876 bp), and a small single-copy region (SSC 20,693 bp). Plastid genome contains 136 genes, 88 protein-coding genes, 38 tRNA genes, and 8 rRNA genes. What is more, a maximum-likelihood phylogenetic analysis demonstrated that *T. cordifolia* was most closely related to *Oberonia japonica* and *Dendrobium salaccense.* The cp genome will provide reference for the further investigation and research of *T. cordifolia*.

Orchidaceae is one of the largest family of monocotyledons (Cameron et al. 1999). It not only has a long history of cultivation and numerous varieties, but also has ecological value. *Tainia cordifolia* is a terrestrial herb belongs to the Orchidaceae family, growing in the evergreen broad-leaved forest at 580–1900 meters above sea level or in the mountain stream (Chen and Wood 2009). Therefore, we reported the complete chloroplast genome (cp) of *T. cordifolia* based on Illumina pair-end sequencing data, which would be helpful for its evolution and genetics research.

The leaf sample was collected from Gushan Mountains (26°05′28″N, 119°38′63″E), Fuzhou city, Fujian province, China. The specimen was stored at Fujian Agriculture and Forestry University (specimen code FAFU08457). The total genomic DNA was extracted from fresh leaves according to the methods described by Doyle and Doyle (1987) and sequencing was carried out by the Illumina pair-end technology. Raw reads were filtered using NGS QC Toolkit (Patel and Jain 2012). Clean reads were first aligned to *Calanthe triplicata* (GenBank Accession No. NC_024544) and *Calanthe davidii* (GenBank Accession No. NC_037438). Filtered reads were then assembled into contigs in the software Platanus version 1.2.4 (Kajitani et al. 2014). The physical map of the new chloroplast genome was generated using OGDRAW (Lohse et al. 2013). Finally, the validated complete cp genome sequence was submitted to GenBank with accession number MN577470.

The complete cp genome of *T. cordifolia* is 158,089 bp in length, containing a large single-copy (LSC) region of 86,876 bp, a small single-copy (SSC) region of 20,693 bp, and two inverted repeat (IR) regions of 25,260 bp. The new sequence has a total of 136 genes, including 88 protein-coding genes, 38 tRNA genes, and 8 rRNA genes. The overall GC-content of the whole plastome is 37.3%, whereas the corresponding values of the LSC, SSC, and IR regions are 54.95%, 13.09%, and 15.98%, respectively.

To further investigate its phylogenetic position, 12 complete cp genomes of Orchidaceae (*Cattleya crispata*, *Cattleya liliputana*, *Masdevallia picturata*, *Corallorhiza odontorhiza*, *Oncidium* ‘Gower Ramsey’, *Erycina pusilla*, *Cymbidium tortisepalum*, *Calanthe triplicata*, *Calanthe davidii*, *Tainia cordifolia*, *Oberonia japonica*, *Dendrobium salaccense*) were aligned using HomBlocks pipeline (Bi et al. 2018). All the data were downloaded from NCBI GenBank. RAxML-HPC2 on XSEDE version 8.2.10 (Stamatakis 2014) was used to construct a maximum likelihood tree. The ML tree analysis indicated that *Oberonia japonica* and *Dendrobium salaccense* closely related to *T. cordifolia* with 100% bootstrap support (Figure 1).

**Figure 1.**
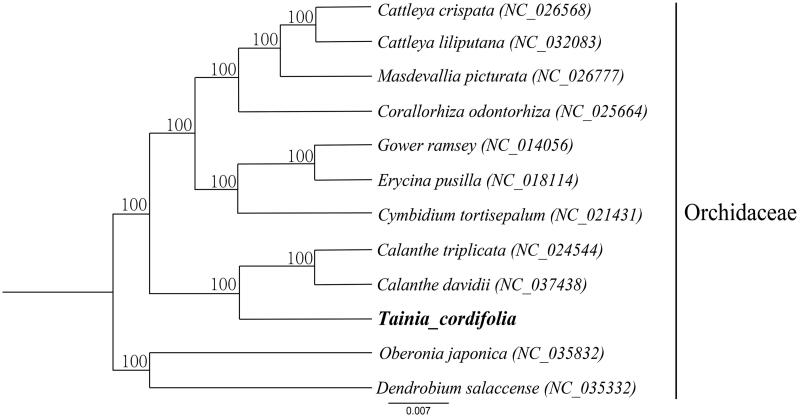
A phylogenetic tree was constructed based on 12 complete chloroplast genome sequences of Orchidaceae. All the sequences were downloaded from NCBI GenBank.
